# Impacts of Climate Change on Rice Grain: A Literature Review on What Is Happening, and How Should We Proceed?

**DOI:** 10.3390/foods12030536

**Published:** 2023-01-25

**Authors:** Ling Tang, Aoqi Wu, Shenshen Li, Mairemu Tuerdimaimaiti, Guoyou Zhang

**Affiliations:** 1 School of Management Science and Engineering, Nanjing University of Information Science and Technology, Nanjing 210044, China; 2 Key Laboratory of Agrometeorology of Jiangsu Province, School of Applied Meteorology, Nanjing University of Information Science and Technology, Nanjing 210044, China; 3 Jiangsu Key Laboratory of Crop Genetics and Physiology, Agricultural College, Yangzhou University, Yangzhou 225000, China

**Keywords:** atmospheric change, carbon dioxide, high temperature, grain quality, co-citation, citation burst

## Abstract

More than half of the people on Earth get their calories, proteins, and minerals from rice grains. Staple increases in the quantity and quality of rice grains are key to ending hunger and malnutrition. Rice production, however, is vulnerable to climate change, and the climate on Earth is becoming more fluctuating with the atmospheric change induced by human activities. As a result, the impacts of climate change on rice grain (ICCRG) have sparked widespread concern. In order to reveal the development and the trend in the study on the ICCRG, a bibliometric analysis was conducted. The results showed that both the model simulations and the field experiment-based observations, as reflected by APSIM (the Agricultural Production Systems sIMulator) and free-air carbon dioxide (CO_2_) enrichment, are of concern to researchers worldwide, especially in China, India, the United States, and Japan. Different types of warming include short-term, nighttime, soil and water, and canopy, and their interactions with other climate factors, such as CO_2_, or agronomic factors, such as nitrogen level, are also of concern to researchers. Spatiotemporal variations in changing weather and regional adaptations from developed and developing countries are challenging the evaluation of ICCRG from an economic perspective. In order to improve the efficacy of breeding adaptable cultivars and developing agronomic management, interdisciplinary studies integrating molecular biology, plant physiology, agronomy, food chemistry, ecology, and socioeconomics are needed.

## 1. Introduction

Since the COVID-19 pandemic outbreak, the number of hunger-affected people has increased by around 150 million; between 2019 and 2020, it increased by 103 million; and in 2021, it increased by 46 million [1]. The prevalence of undernourishment increased from 8.0 to 9.3 percent from 2019 to 2020, then increased more slowly in 2021 to 9.8 percent after being largely stable since 2015. 

Rice (*Oryza sativa* L.) is one of the most important crops on Earth. More than 100 countries grow rice, a grain that is a major food supply for more than half of the world’s population [2,3,4,5,6]. Rice grain is a rich source of calories, magnesium, phosphorus, manganese, selenium, iron, folic acid, thiamin, and niacin, but it is also a good source of these nutrients [7,8,9,10]. Thus, the changes in the quantity and quality of rice grain will affect global food security, which is, unfortunately, vulnerable to climate change.

The impacts of climate change are placing strain on agriculture and making it harder to produce enough food [11]. Both direct and indirect effects of climate change, such as altered precipitation patterns, droughts, flooding, and the spatial distribution of pests and diseases, have an impact on the productivity of agriculture. It has been assessed that the rise of agricultural output in mid- and low-latitude areas has been inhibited by human-induced climate change, at least over the past 50 years [12]. Increasing salinity in the cultivable regions can make farming riskier as a result of rising temperatures, which results in a rise in sea level. Additionally, both the macro- and microenvironmental effects of extreme weather events on crop growth are present in rice, which is particularly susceptible to a rise in frequency and severity. For example, the rising concentration of atmospheric carbon dioxide (CO_2_) has a fertilizer effect on plant photosynthesis and water use efficiency, which can increase the quantity of grains in rice cultivars but degrade the quality of the grains [13,14,15,16]. However, the CO_2_ fertilization effects can be easily offset by other climate factors, such as warming [17]. Rising concentrations of surface ozone (O_3_) also have essential impacts on the quantity and quality of rice grains [18,19,20].

Although numerous studies on the impacts of climate change on crops have been conducted, the foci and hotspots of the research on the impacts of climate change on rice grains (ICCGR) have not been clarified yet. In order to clarify the most concerning themes, which involves areas, international distributions, and collaborations in ICCRG studies, this bibliometric analysis was conducted.

## 2. Materials and Methods

### 2.1. Data Sources and Screening

The Web of Science Core Collection (WoSCC) database was used in numerous bibliometric studies and is well-suited to this type of analysis. As a result, all reference data for our study were obtained from the WoSCC, including Clarivate Analytics’ Science Citation Index Expanded (SCIE) studies. The search was conducted on 21 November 2022 to collect academic publications from WoSCC, using (all fields (climate change) AND all fields (rice) AND all fields (grain)) and obtaining 1497 records about studies on the ICCRG. Todhunter PE et al. [21] and Terjung W et al. [22] published the first article on ICCRG research, which can be retrieved from WoSCC, in 1989. As a result, the time span for data retrieval was 1989–2022.

### 2.2. Analytical Methods

Due to the large number of publications identified, manually extracting their information would be prohibitively difficult, necessitating the use of software. CiteSpace is a Java application that analyzes and visualizes co-citation networks [23,24], including co-citation references, co-authors, and co-occurring keywords, to aid in the delivery of ICCRG knowledge domain results. As a result, we chose CiteSpace version 6.1R3 (http://cluster.ischool.drexel.edu/cchen/citespace/download/, accessed on 11 November 2022) as the primary tool to provide a thorough analysis of the selected literature. 

CiteSpace was utilized to look into the distribution and cooperation across nations, research institutes, and writers, as well as the networks of publications and collaboration. By modifying the node types in the CiteSpace program to “country,” “institution,” and “author,” correspondingly, we were able to achieve this. Due to their tight ties, we included the country and institution nodes in the same figure (i.e., institutions are subsets of a country). By simultaneously setting node types to “category” and utilizing the software’s timeline view, we were able to depict the development of study subjects in this area. By simultaneously setting node types to “category” and utilizing the software’s timeline view, we were able to depict the development of study subjects in this area. Co-citation analysis was possible when the node types were set to “reference.” (In this study, we defined a co-citation as an instance in which papers A and B simultaneously quote paper C.) 

Using co-citation analysis provided by the CiteSpace software, we developed a knowledge base for research in this area and determined the most significant citations. Using this information, we carried out a number of dynamic change assessments to determine how the knowledge base changed. Using the co-occurrence of “terms” and cluster analysis, we were able to pinpoint cutting-edge research and hot regions at different stages of the field’s development. By altering the node type to “Terms” and clustering the nodes in the term co-occurrence graph, the graphs that reflected these studies were produced. By taking into consideration the findings of all these investigations, we made predictions about future issues and scientific advances.

## 3. Results

### 3.1. The Developing Concern with the ICCRG

**Figure 1:**
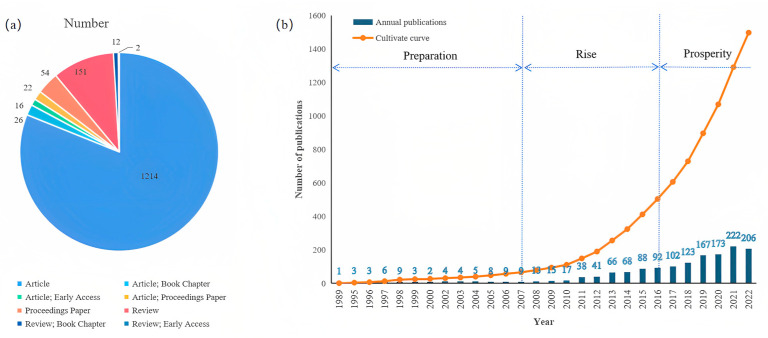
The annual publication volume of ICCRG research: (**a**) number and type of articles (retrieval date: 22 November 2022); (**b**) annual volume and cumulative volume of publications.

There were 1497 papers assessed, all of which were written between 1989 and 2022. The ICCRG research is expanding exponentially right now, according to the published trend (Figure 1). After examining the individual titles and abstracts, 1497 pertinent papers (1332) and reviews (165) covering the years 1993 to 2022 were found (Figure 1a). According to the number of published articles, there were three distinct stages (Figure 1b). The number of papers published during the 14 years from 1989 to 2007 accounted for only 4.41% of the total, and there were no more than 10 papers published in any year during this stage. Although the research at this stage was not abundant, acceptance of the ICCRG and its research methods established a theoretical basis for subsequent research. As a result, we refer to these years as the ICCRG research’s “preparation” stage.

The number of papers published in the ICCRG increased dramatically between 2007 and 2016, reaching 6.64 times the 2007 total by the conclusion of this time frame and making up 29.26% of the publications during our study period. This phase of the ICCRG is known as the “raising” phase. Throughout this period, the ICCRG’s research continued to advance and develop. Since 2017, more than 100 papers have been published annually, marking the start of the ICCRG study’s “prosperity stage.” Since 66.33% of the total number of publications during this time period were in this area, the ICCRG developed into a popular area of study for many academics.

CiteSpace statistics showed that the 1497 publications we examined cited 44,075 references. Despite the fact that our literature search turned up studies from as early as 1989, there were not enough publications until 2000 to create clusters. Through co-citation analysis, the most concerning themes involving areas and international distributions and cooperations in the studies on ICCRG were revealed.

### 3.2. The concerning Themes in Studying the ICCRG

#### 3.2.1. Themes Reflected by Keyword Clusters

**Figure 2:**
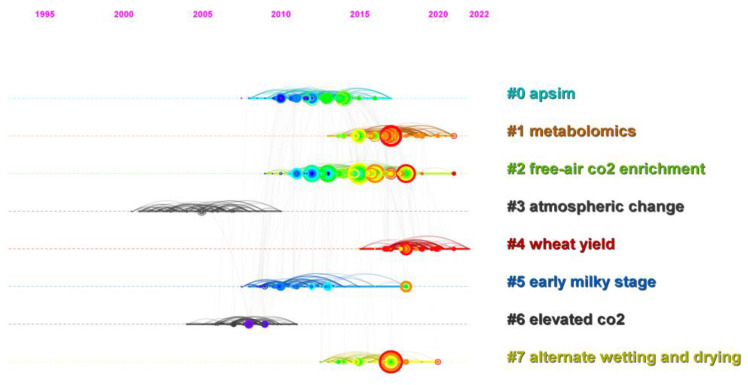
Timelines of keyword clusters for co-cited references. Major clusters are labeled on the right. The colors were used to improve the visibility.

Eight major clusters were found that represented the body of knowledge for ICCRG research after grouping the referenced articles to establish the top 23 keyword clusters (based on their frequency) in each year, reflecting the major themes in this field. They were “apsim”, “metabolomics”, “free-air co2 enrichment”, “atmospheric change”, “wheat yield”, “early milky stage”, “elevated co2”, and “alternate wetting and drying”, in order of frequency (Figure 2).

The “apsim” was the most frequent theme in studying the ICCRG, especially from 2008 to 2017. The “metabolomics” cluster was the last to form at the time of our investigation, starting in 2013 and lasting until 2022. It somewhat reflected the current ICCRG focus shift toward molecular biology. The “free-air co2 enrichment” cluster, which emerged from 2009 to 2021, was the largest, comprised the most referenced papers, and lasted the longest (12 years), reflecting the field’s attention on the study’s topic. The term “atmospheric change” first appeared in articles published in 2000. The majority of the mentioned papers dealt with climate change. The “wheat yield” (2015–2022) reflected that interaction among crops. The “early milky stage” cluster, which had an emphasis on crop physiology, lasted for a lengthily period of time (about 2007–2018). The “elevated co2” and “alternate wetting and drying” emerged from 2004 to 2011 and from 2012 to 2020, respectively. 

#### 3.2.2. Themes Reflected by Item Clusters 

**Figure 3:**
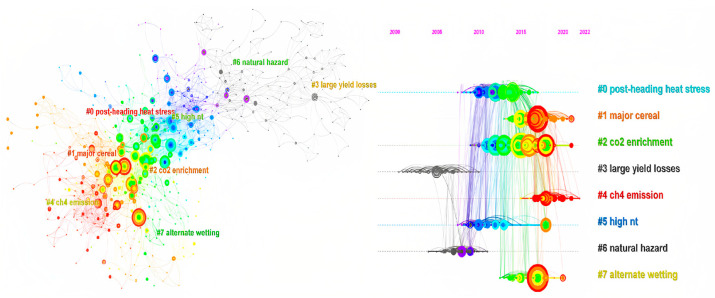
Item (title, keyword, and abstract) clusters for co-cited references. Major clusters are labeled on the right. The colors were used to improve the visibility.

Eight top themes were identified by clustering the tile, keyword, and abstract of co-cited references (Figure 3).

Post-heading heat stress, the largest cluster (#0), had 89 members and a silhouette value of 0. It was labeled as post-heading heat stress by log-likelihood ratio (LLR), heat stress by Latent Semantic Indexing (LSI), and high production cost (1.53) by mutual information (MI). The major citing article of the cluster was “Rice responses to rising temperatures—challenges, perspectives, and future directions” [25], and the most-cited members of the cluster were “Extreme heat effects on wheat senescence in India” [26], “Temperatures and the growth and development of maize and rice: a review” [27], and “Producing more grain with lower environmental costs” [28].

Major Cereal was the second largest cluster (#1), with 89 members and a silhouette value of 0. LLR classified it as a major cereal, LSI classified it as high temperature, and MI classified it as high production cost (1.65). The major citing article of cluster 1 was “The heat is on: how crop growth, development, and yield respond to high temperature” [29], and the most-cited members of the cluster were “Temperature increase reduces global yields of major crops in four independent estimates” [30], “Post-flowering night respiration and altered sink activity account for high night temperature-induced grain yield and quality loss in rice (*Oryza sativa* L.)” [31], and “Influence of extreme weather disasters on global crop production” [32].

CO_2_ enrichment was the third largest cluster (#2) and had 75 members and a silhouette value of 0. It was labeled as CO_2_ enrichment by LLR, rice yield by LSI, and high production cost (2.52) by MI. The major citing article of cluster 2 was “Rice grain yield and quality responses to free-air CO_2_ enrichment combined with soil and water warming” [33], and the most-cited members of the cluster were “Uncertainties in predicting rice yield by current crop models under a wide range of climatic conditions” [34] and “Responses of wheat and rice to factorial combinations of ambient and elevated CO_2_ and temperature in FACE (free-air CO_2_ enrichment) experiments [35] and global warming of 1.5 °C” [36]. 

Large Yield Losses was the fourth largest cluster (#3), with 73 members and a silhouette value of 0. It was labeled as large yield losses by LLR, wet season by LSI, and fossil-fuel greenhouse gas (0.08) by MI. The major citing article of cluster 3 was “Global food insecurity: treatment of major food crops with elevated carbon dioxide or ozone under large-scale, fully open-air conditions suggests recent models may have overestimated future yields” [37], and the most-cited members of the cluster were “What have we learned from 15 years of free-air CO_2_ enrichment (FACE)? A meta-analytic review of the responses of photosynthesis, canopy properties, and plant production to rising CO_2_” [38] and “Summary for Policymakers [39] and Food for Thought: Lower-Than-Expected Crop Yield Stimulation with Rising CO_2_ Concentrations” [40]. 

CH4 Emission was the fifth largest cluster (#4), with 69 members and a silhouette value of 0. LLR labeled it as CH4 emission, LSI as degree C, and MI as high production cost (1.17). The major citing article of cluster 4 was “Effects of free-air temperature increase on grain yield and greenhouse gas emissions in a double rice cropping system” [41], and the most-cited members of the cluster were “Impacts of climate change on rice production in Africa and causes of simulated yield changes” [42], “Do all leaf photosynthesis parameters of rice acclimate to elevated CO_2_, elevated temperature, and their combination in FACE environments?” [43], and “Higher yields and lower methane emissions with new rice cultivars” [44]. 

HIGH NT (high nighttime temperature) was the sixth largest cluster (#5), with 61 members and a silhouette value of 0. It was labeled as high NT by LLR, high heat stress by LSI, and high production cost (0.59) by MI. The major citing article of cluster 5 was “Rice responses to rising temperatures—challenges, perspectives, and future directions” [25], and the most-cited members of the cluster were “Physiological and proteomic approaches to address heat tolerance during anthesis in rice (*Oryza sativa* L.)” [45], “Summary for Policymakers of IPCC Special Report on Global Warming of 1.5 °C approved by governments” [46], and “Rice yields in tropical/subtropical Asia exhibit large but opposing sensitivities to minimum and maximum temperatures” [47].

Natural Hazard was the seventh largest cluster (#6), with 56 members and a silhouette value of 0. It was labeled as a natural hazard by LLR, a low grain yield by LSI, and a high production cost (0.25) by MI. The major citing article of cluster 6 was “High-temperature effects on rice growth, yield, and grain quality” [48], and the most-cited members of the cluster were “Prioritizing Climate Change Adaptation Needs for Food Security in 2030” [49], “Rice production in a changing climate: a meta-analysis of responses to elevated carbon dioxide and elevated ozone concentration” [50], and “Climate change affecting rice production: the physiological and agronomic basis for possible adaptation strategies” [51].

Alternate Wetting was the eighth largest cluster (#7), with 56 members and a silhouette value of 0. It was labeled as alternate wetting by LLR, water productivity by LSI, and high production cost (0.7) by MI. The major citing article of cluster 7 was “Alternate wetting and drying in Bangladesh: water-saving farming practice and the socioeconomic barriers to its adoption” [52], and the most-cited members of the cluster were “Rice yields and water use under alternate wetting and drying irrigation: A meta-analysis” [53], “Reducing greenhouse gas emissions, water use, and grain arsenic levels in rice systems” [54], and “Effects of water-saving irrigation practices and drought resistant rice variety on greenhouse gas emissions from a no-till paddy in the central lowlands of China” [55].

#### 3.2.3. Themes Reflected by Keyword Burst

**Figure 4:**
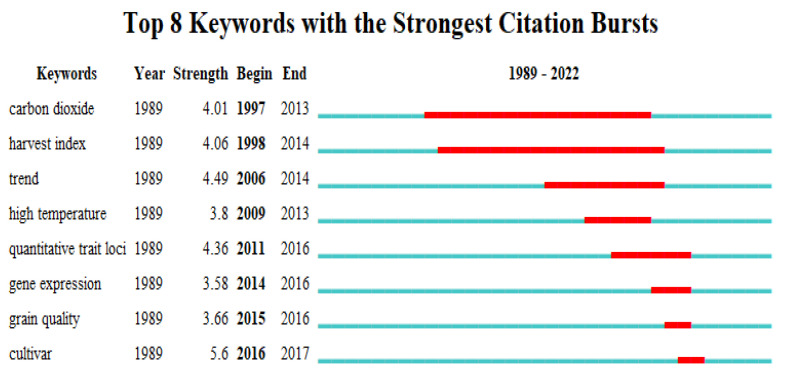
Keywords with the strongest citation bursts. The bule and red lines indicate the emergence of keywords from 1989 to 2022 and the durations with the strongest citation bursts, respectively.

It is important to pinpoint the significant rises in interest and the study frontiers of a specific specialty based on the burstiness of keywords. No matter how often their host articles are cited, CiteSpace is able to identify developing keywords [56]. In this instance, eight burst keywords (Figure 4) were found. The majority of the burst keywords were produced after 1997, indicating that around this time, the ICCRG attracted significant attention and began to diversify. The top keyword associated with the climate change burst, which began in 1997 and lasted until 2013, was “carbon dioxide”, followed by “harvest index”, which began in 2018 and lasted until 2014. The third top keyword was “trend”, which lasted from 2006 to 2014, followed by “high temperature”, “quantitative trait loci”, “gene expression”, “grain quality”, and “cultivar”, which lasted from 2009 to 2013, 2011 to 2016, 2014 to 2016, 2015 to 2016, and 2016 to 2017, respectively.

### 3.3. Involving Areas in the Studies on ICCRG

**Figure 5:**
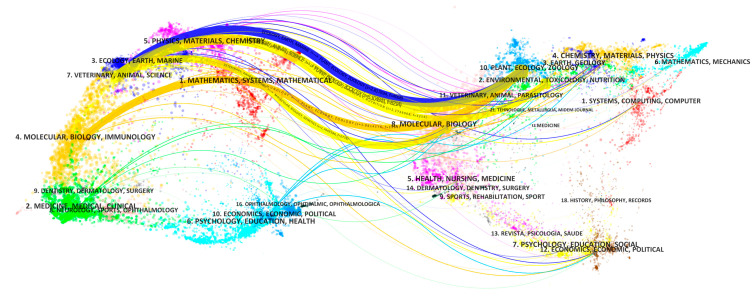
A dual-map overlay of the 1497 publications on ICCRG research. The colors were used to improve the visibility.

A dual-map overlay of ICCRG publications published between 1989 and 2022 is shown in Figure 4. The pathways of the citation linkages all are represented by colored arcs, leading from the citing map and directed to the cited map. Thematic areas based on publishing journals were divided into citing and cited maps, and each area was labeled with the most prevalent words in the titles of relevant articles. The fields in which the cited papers were written are shown on labels next to the launch zones. The literature on ICCRG research can be found in a number of areas, as shown in Figure 5.

The top section was colored purple and was labeled physics/materials/chemistry; the top section was blue and was labeled ecology/earth/marine; the middle section was yellow and was labeled veterinary/animal/science; the middle section was orange and was labeled molecular biology/biology/immunology; and the bottom section was green and was labeled medicine/medical/clinical. However, the majority of these cited publications were in the molecular biology/immunology and ecology/earth/marine fields. Additionally, the majority of the mentioned publications were published in journals devoted to molecular biology, genetics, environmental toxicology, zoology, ecology, and plants.

### 3.4. International Distributions and Collaborations in ICCRG Studies

**Figure 6:**
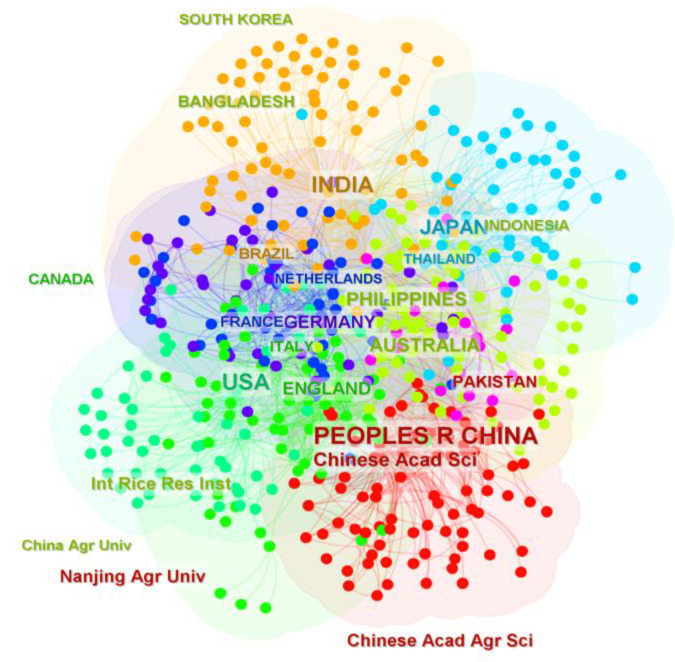
Map of the cooperation networks among countries and institutions in the field of ICCRG. Nodes with count 30 (there are 30 or more publications in a country and institution) are tagged with names. The colors were used to improve the visibility.

It is possible to identify the important nations and research institutions that created a significant number of publications and grew to have a significant impact on the field of ICCRG, as well as the cooperative relationships between them, by analyzing the network of cooperation among nations and institutions. We identified 470 institutions that engaged in research on the ICCRG across 100 nations or regions, with 19 countries and 6 institutions producing the most articles (Figure 6). The top three countries in terms of publications were China (464), India (282), and the United States (261), yet these three had quite different institutional distributions.

Chinese Acad Sci (Chinese Academy of Science), Nanjing Agr Univ (Nanjing Agriculture University), Chinese Acad Agr Sci (Chinese Academy of Agriculture Sciences), Univ Chinese Acad Sci (University of Chinese Academy Science), and China Agr Univ (China Agriculture University) were just a few of the major research institutions where the majority of China’s research output was concentrated. The Chinese Academy of Sciences stood out in particular, producing 111 publications, more than the Philippines, which was ranked fifth in terms of the overall number of publications it produced (104). ICCRG research institutions were more widespread in the United States and India, but neither country produced more than 30 articles in total.

The degree of centrality is crucial from the viewpoint of a cooperative network. A node’s centrality indicates how strong it is in the overall network based on the number of connections it has to other nodes; a node with a high centrality is a critical node with a significant impact on network relationships. Critical nodes in CiteSpace are nodes with an intermediary centrality of greater than 0.1. India had the highest level of centrality ((Centr) = 0.164), followed by the US ((Centr) = 0.149), Japan ((Centr) = 0.102), and China ((Centr) = 0.100). Additionally, there was tight collaboration between those nations. These nations also worked closely with the Philippines, Austria, France, Germany, Thailand, Vietnam, Laos, Indonesia, Malaysia, and other nations that cultivate rice.

## 4. Discussion

### 4.1. What Is Happening?

#### 4.1.1. Rising Concentrations of Atmospheric Carbon Dioxide and the Fertilization Effect

According to the stated trend, ICCRG research is now growing rapidly (Figure 1). The most concerning theme in ICCRG is carbon dioxide (Figure 4), the effects of rising concentrations of atmospheric carbon dioxide (Figure 3). According to research from NOAA’s Worldwide Monitoring Lab, despite the ongoing economic impact of the COVID-19 pandemic, the average global atmospheric carbon dioxide level in 2021 was 414.72 parts per million (ppm), a new record high [57]. Thus, according to NOAA’s 63-year record, the increase of 2.58 ppm over 2021 matched the fifth-highest yearly increase. Despite the greenhouse gas effect, rising levels of atmospheric carbon dioxide acted as fertilizer for plants and crops [58,59,60,61], a phenomenon known as the CO_2_ fertilization effect (CFE). Conversely, CFE minus the quality of grains is called the dilution effect. More starch lowers protein, acid, and other nutrient concentrations. Maximizing the CFE to improve crop yield and maintaining or improving the grain quality are all research efforts, and numerous studies on rice have been conducted [62,63,64,65,66].

#### 4.1.2. Field-Experiment-Based Observations and Model Simulations

Field-based experimental studies revealed the variations among different rice cultivars. Grain yield enhancement by CFE varied between rice cultivars, ranging from 3% to 36% [67,68], suggesting the potential of maximizing yield through cultivar screening. Free-air CO_2_ enrichment (FACE) [69], a field simulation system that allowed us to conduct open field experiments, was one of the host themes (Figure 2). Due to the development of the simulation facility, studies on the interactions of elevated concentrations of CO_2_ with other climatic and agronomic factors were also conducted, revealing that the CFE on grain yield was vulnerable. CFE on rice is limited by climatic factors, including the salinity levels of the paddy [70], cool weather [71], warming [33], and the concentration of surface ozone [50]. CFE is also affected by agronomic factors, including nitrogen fertilization levels [72,73], water availability [74], crop rotation [75], etc. Rising CO_2_ levels encourage carbon gain in rice [76] and alley lodging [77], altering dry matter production and distribution [78] and thus the harvest index, which is the ratio of harvestable grain to aboveground biomass and the secondary keyword with the strongest citation bursts (Figure 4). 

Despite the field experiment, studies on the ICCRG through model simulations are also concerning. The “apsim” is the most frequently occurring theme in research on the ICCRG, particularly between 2008 and 2017 (Figure 2). An extremely sophisticated framework for modeling and simulating agricultural systems is known as APSIM [79], or the Agricultural Production Systems sIMulator. It includes a number of modules that make it possible to simulate various plant, animal, soil, climatic, and management interactions. The use of APSIM is employed to look into the potential effects of climate change (including CO_2_, temperature, solar radiation, and precipitation) on crop phenology, yield, and water consumption for rice [80,81,82,83]. However, there is a variation among rice models in yield response to climate change, as measured by field-based experiments [84,85].

#### 4.1.3. Global Warming and Extreme Weather as Reflected by Different Types of Temperature Increases

Warming, as depicted by the post-heading heat stress, high nt (high nighttime temperature) in Figure 3, and high temperature in Figure 4, is also one of the ICCRG’s most concerning themes. Temperature increases in the soil and water in rice paddies, as well as in the rice canopy, decrease the CFE on grain yield, as revealed by FACE studies [17,33]. In addition, even while the mean temperature rises, there isa bigger challenge, due to increasing variability and a faster rise in nighttime temperatures than in daytime maximums [25,86,87]. Both warming and CFE alter grain filling in rice; thus, post-heading heat stress (Figure 3) and the early milky stage (Figure 2) are concerning themes. Given that the duration of grain filling, post-flowering senescence, changes in the starch and protein content of rice grains, starch metabolism enzymes, and chalk formation in rice grains are sensitive to warming [31,88,89], field-observed experiments and model simulation studies are required to identify and breed tolerant cultivars.

#### 4.1.4. Interdisciplinary Studies on the ICCRG

As shown by the dual-map overlay of ICCGR publications (Figure 5), the domains of molecular biology/immunology, ecology/earth/marine, and these articles were the ones most frequently cited. Furthermore, the majority of the cited studies appeared in zoological, botanical, molecular, genetic, environmental, toxicological, and ecological journals. Both the quantity and quality of rice grains are determined by the process of grain filling or grain growth at the point of the rice plant, which is determined by both genetic and environmental factors. Thus, ICCRG studies involve multiple areas, including plant physiology, agronomy, ecology, environmental sciences, socio-economic sciences, etc. [88,89,90,91,92]. There is a trend in the studies on ICCRG shifting toward molecular biology for breeding tolerant cultivars, as shown by “metabolomics” in Figure 2 and “quantitative trait loci” and “gene expression” in Figure 4.

### 4.2. How Should We Proceed?

The many uncertainties surrounding climate change make the role of rice as a staple food for half of the world’s population all the more important. The most urgent need is to better understand ICCRG and its mechanisms. This requires international cooperation, and an economic assessment of ICCRG can strengthen public awareness.

#### 4.2.1. Field Observations with Improved Experiential Design Should Be Conducted

Extreme weather events, such as heat, are becoming more common as a result of global climate change [93,94,95]. Previous field-experiment-based studies on the ICCRG investigated the short-term (acute), long-term (chronic), seasonal, and multiple-year effects of climate factors [14,20,78]; however, the investigated climate factors were limited [12]. Although the interaction effects between carbon dioxide and temperature [17], drought [96], ozone [97,98,99], and nitrogen [100,101] on rice were investigated, interactions with other climate factors remain to be investigated. The FACE scale makes it possible to research both physiology and psychology simultaneously, which can succumb to disease and soil processes [69], though there are only a few FACE studies on rice in China, Japan, and India. Additionally, FACE studies should be encouraged, especially in rice-planting countries where the local rice cultivars may maintain key information for breeding new, adaptable varieties.

#### 4.2.2. International Cooperation Should Be Strengthened

Deep international cooperation will be necessary for the ICCRG study and effective climate change mitigation [102,103]. The ICCRG was studied by 470 institutions from 100 different countries or regions (Figure 6); China, Japan, the United States, and India collaborated closely. Out of 470 institutions from 100 different nations or regions, 19 institutions produced the most articles, followed by 6 institutions. China, India, and the United States were first through third in terms of publications, but their institutional distributions were very different. ICCRG research institutions were concentrated in China but widely spread in the United States and India. China, Japan, the United States, and India also collaborated closely with the Philippines, Austria, France, Germany, and other rice-growing countries in Asia. ICCRG studies in Africa, however, are limited and need to be improved [42]. 

#### 4.2.3. ICCRG from the Economic Perspective

The increasing, extreme weather events are challenging the evaluation and prediction of the ICCRG, especially from the economic perspective [104,105,106]. This is because aside from the variations in the yield loss evaluation, the impact of climate change on the grain quality traits is usually underestimated, lowering the economic loss induced by climate change [107,108]. Spatiotemporal variations in changing weather factors, including temperature and precipitation, affect the distribution of agricultural production, food supply, and world markets. Not all regions will suffer economic losses from climate change because, in the low–medium temperate zone, positive economic effects can be realized due to the comparative advantage from differences in labor productivity that change between regions [109,110,111,112,113,114]. However, food systems are proving more vulnerable as agricultural trade networks become more centralized, and a few regions dominate markets under climate change [115,116,117,118]. ICCRGs in developed and developing countries are different, due to the different tolerances of their agricultural production and food trade systems to climate change and extreme weather events [119,120,121]. Global climate change, regional extreme weather events, the global distribution of food supply systems, and adaptation actions in developed and developing countries have limited the ICCRG’s assessment of its economic prospects, and the future requires integrated research.

## 5. Conclusions

Through this bibliometric analysis, the most concerning themes, which involved areas, international distributions, and collaborations in ICCRG studies, were clarified. The most concerning themes included carbon dioxide and warming as climatic factors; grain yield, grain quality, and grain growth (early milky stage) as rice traits; and quantitative trait loci, gene expression, and alternate wetting as breeding and agronomic adaptations. ICCRG studies involved multiple areas, including plant physiology, agronomy, ecology, environmental sciences, and socio-economic sciences. China, India, and the United States ranked first through third in publications promoting the study of the ICCRG. 

## Data Availability

The data are available from the corresponding author.

## Abbreviations

ICCRG (the impacts of climate change on rice grain); APSIM (The Agricultural Production Systems sIMulator); CO_2_ (carbon dioxide); WoSCC (The Web of Science Core Collection); SCIE (Science Citation Index Expanded); LLR (log-likelihood ratio); LSI (Latent Semantic Indexing); MI (mutual information); FACE (free-air CO_2_ enrichment); Chinese Acad Sci (Chinese Academy of Science); Nanjing Agr Univ (Nanjing Agriculture University); Chinese Acad Agr Sci (Chinese Academy of Agriculture Sciences); Univ Chinese Acad Sci (University of Chinese Academy Science); China Agr Univ (China Agriculture University); and CFE (CO_2_ fertilization effect).
